# Protective effects of quercetin and taraxasterol against H_2_O_2_-induced human umbilical vein endothelial cell injury *in vitro*

**DOI:** 10.3892/etm.2015.2713

**Published:** 2015-08-25

**Authors:** DONGWEI YANG, XINYE LIU, MIN LIU, HAO CHI, JIRONG LIU, HUAMIN HAN

**Affiliations:** 1 Department of Cardiology, Zhengzhou Central Hospital Affiliated to Zhengzhou University, Zhengzhou, Henan 450007, P.R. China; 2 Department of Internal Medicine, North China Electric Power University Hospital, Beijing 102206, P.R. China

**Keywords:** atherosclerosis, quercetin, taraxasterol, anti-inflammatory, human umbilical vein endothelial cells

## Abstract

Due to the association between inflammation and endothelial dysfunction in atherosclerosis, the blockage of the inflammatory process that occurs on the endothelial cells may be a useful way of preventing atherosclerosis. In the present study, human umbilical vein endothelial cells (HUVECs) were used to investigate the protective effects of quercetin and taraxasterol against H_2_O_2_-induced oxidative damage and inflammation. HUVECs were pretreated with quercetin or taraxasterol at concentrations ranging between 0 and 210 µM for 12 h, prior to being administered different concentrations of H_2_O_2_ for 4 h. Cell viability and levels of apoptosis were assessed through cell counting kit-8 (CCK-8) and terminal deoxynucleotidyl transferase dUTP nick end labeling assays, respectively, to determine the injury to the HUVECs. The viability loss in the H_2_O_2_-induced HUVECs was markedly restored in a concentration-dependent manner by pretreatment with quercetin or taraxasterol. This effect was accompanied by significantly decreased expression of vascular cell adhesion molecule 1 (VCAM-1) and cluster of differentiation (CD)80 for taraxasterol and that of CD80 for quercetin. In conclusion, the present study showed the protective effects of quercetin and taraxasterol against cell injury and inflammation in HUVECs and indicated that the effects were mediated via the downregulation of VCAM-1 and CD80 expression. This study has therefore served as a preliminary investigation on the anti-atherosclerotic and cardiovascular protective effects of quercetin and taraxasterol as dietary supplements.

## Introduction

Atherosclerosis is a complex process with multiple risk factors. Accumulating evidence suggests that endothelial cell dysfunction initiates and accelerates the development and progression of atherosclerosis, and plays a pivotal role in this process. In the past decade, endothelial dysfunction has been used as a target for the prevention of cardiovascular disease (1). The association between inflammation and endothelial dysfunction has been shown in metabolic syndrome, diabetes, inflammatory bowel disease and experimental hypertension (2,3). Furthermore, inflammation has been identified as an independent risk factor for cardiovascular diseases, and has a major role in all phases of atherosclerosis (4,5). Vascular inflammation is thus a key factor in the initiation and progression of atherosclerotic plaque. Atherosclerosis is an inflammatory process that may result in the formation of unstable coronary lesions vulnerable to disruption and subsequent thrombosis. Anti-inflammatory treatment strategies may improve endothelial function and play an important role in the prevention and treatment of atherosclerosis.

The overexpression of pro-inflammatory cellular adhesion molecules (CAMs) has been reported in the cardiovascular system of animal models of atherosclerosis (6). CAMs are involved in the recruitment of inflammatory cells, cell-cell and cell-matrix interactions and signal transduction within the developing atherosclerotic lesion. Furthermore, patients with carotid artery atherosclerosis have been found to exhibit enhanced circulating levels of soluble E-selectin and intracellular adhesion molecule 1 (ICAM-1) (7), which may serve as molecular markers for atherosclerosis. Systemic levels of ICAM-1 and VCAM-1 are associated with intimal-medial thickness and the degree of atherosclerosis in hypertensive type-2 diabetic patients (8). Several anti-inflammatory interleukins (ILs), however, have been suggested to possess anti-atherosclerotic effects, including IL-4, IL-10, IL-19 and IL-33 (9–11).

Statins have demonstrated not only an inhibition of plasma lipid levels, but also anti-inflammatory and antioxidant activities on endothelial cells, resulting in the beneficial reduction of atherosclerotic processes and cardiovascular risk in animal models and humans (12–15). These effects are believed to occur through the suppression of monocytes and the expression of ICAM-1, monocyte chemoattractant protein-1, IL-8, IL-6 and cyclooxygenase-2 (16). Of note, the protective effect of high-density lipoprotein against the development of atherosclerosis may be partly due to its anti-inflammatory and antioxidant properties. Since inflammation contributes to the formation and progression of atherosclerosis, certain anti-inflammatory drugs have been evaluated for possible anti-atherosclerotic effects (17). Numerous herbs and supplements possess anti-inflammatory activities, such as curcumin, *Boswellia*, white willow bark and ginger root extracts, bromelain, quercetin and taraxasterol.

Quercetin, a bioflavonoid commonly found in a variety of plants, is known to exert several biological effects, including anti-inflammatory, antiviral and antitumor activities, inhibition of platelet aggregation and adhesion, and vascular endothelial cell-dependent vasodilation. These effects are beneficial in numerous cardiovascular diseases, such as coronary artery disease, hypertension, atherosclerosis and stroke. The highest levels of quercetin are found in plant-based foods, such as apples, onions, berries and red wine. Chronic intake of quercetin can decrease blood pressure (18–20), reduce plasma oxidized low-density lipoprotein (LDL) concentrations in overweight subjects with a high cardiovascular disease risk phenotype (19) and inhibit LDL oxidation (21). Quercetin exerts cardiovascular protective effects (22) and inhibits platelet aggregation and essential components of the collagen-stimulated platelet activation pathway in humans (23). Although a previous study has shown that the flavonoid quercetin protects H_2_O_2_-injured vascular endothelial cells by an antioxidant mechanism (18), much remains unclear about the mechanisms involved in the protection. Taraxasterol, a pentacyclic-triterpene isolated from the Chinese medicinal herb *Taraxacum officinale*, also exhibits an anti-inflammatory effect (24). Taraxasterol has been suggested to perform its cardiovascular protection through the restoration of endothelial cell function (25). To clarify this point, it is necessary to carefully explore and compare the direct contributions of taraxasterol and quercetin to the protection against endothelial dysfunction and the inhibition of the proinflammatory vascular events that initiate the atherosclerotic process. Of note, however, is that the potential interaction between these two agents and inflammatory cytokines, which can promote vascular inflammation, has yet to be fully elucidated and should be taken into consideration. For this purpose, the aim of the present study was to explore the respective effects of taraxasterol and quercetin in H_2_O_2_-induced endothelial injury.

## Materials and methods

### 

#### Chemicals and reagents

H_2_O_2_ was purchased from Sinopharm Chemical Reagent Co., Ltd. (Shanghai, China). 4′,6′-Diamidino-2-phenylindole (DAPI) and paraformaldehyde were obtained from Sigma-Aldrich (St. Louis, MO, USA), and quercetin and taraxasterol were purchased from Sigma (St. Louis, MO, USA). Phycoerythrin (PE)-conjugated anti-human ICAM-1 and anti-human cluster of differentiation (CD)106 (VCAM-1) were obtained from eBioscience (San Diego, CA, USA). Quercetin and taraxasterol were dissolved in dimethyl sulfoxide (DMSO) and diluted to different concentrations by cell culture medium according to the requirement of the experiment (the final concentration of DMSO did not exceed 0.5%). H_2_O_2_ (30%) was diluted into different concentrations by cell culture medium if necessary.

#### Cell viability

Cell viability was assessed using a cell counting kit-8 (CCK-8) assay (Dojindo Laboratories, Kumamoto, Japan). Human umbilical vein endothelial cells (HUVECs; American Type Culture Collection, Rockville, MD, USA) were seeded in 96-well plates at an initial density of 3×10^4^ cells/well. After 12 h of incubation, the cells were treated in the presence or absence of quercetin or taraxasterol for 12 h at concentrations of 0–210 µM (30, 60, 90, 120, 150, 180 or 210 µM), or treated with different concentration of H_2_O_2_ (0, 200, 400, 800 or 1,600 µM) for 4 h. The medium was removed and the cells were washed twice with fresh media. Following washing, 100 µl fresh serum-free Dulbecco's modified Eagle's medium (Gibco Life Technologies, Carlsbad, CA, USA) containing 1/10 (v/v) CCK-8 reagent was added to each well and incubated for an additional 4 h. Following incubation, the viability of the HUVECs was assessed using a 96-well plate reader (DG5032; Huadong, Nanjing, China) at 450 nm. The survival rate of the cells was calculated using the following formula: Survival rate (%) = optical density (OD) of the treated cells - OD of blank control/OD of negative control - OD of blank control x100.

#### Determination of the ability of quercetin and taraxasterol to attenuate the cytotoxic effect of H_2_O_2_

HUVECs were seeded in 96-well plates at an initial density of 3×10^4^ cells/well. After 12 h of incubation, the cells were treated in the presence or absence of quercetin or taraxasterol for 12 h at concentrations of 0–210 µM (30, 60, 90, 120, 150, 180 or 210 µM), followed by the addition of 800 µM H_2_O_2_ for 4 h. The CCK-8 assay was performed as described earlier.

#### Flow cytometric detection of apoptosis and necrosis

Cell death was analyzed by flow cytometry, for which the cells were stained with annexin V labeled with the fluorescent probe fluorescein isothiocyanate (FITC) and propidium iodide (PI). Cells cultured in the absence or in the presence of H_2_O_2_ were incubated for 10 min at 4°C in 440 µl annexin buffer (Immunotech kit; Beckman Coulter, Marseilles, France) containing 5 µl FITC-labeled annexin V and 5 µl PI. The cells were then washed with phosphate-buffered saline and resuspended in the same buffer. The HUVECs were also examined with a fluorescence-activated cell sorting (FACS)-based terminal deoxynucleotidyl transferase dUTP nick end labeling (TUNEL) assay (APO-Direct™ kit; BD Pharmingen, San Diego, CA, USA). Samples were analyzed using a flow cytometer (FACSCalibur; BD Biosciences, Franklin Lakes, NJ, USA) and data were processed using CellQuest software (BD Biosciences).

#### Flow cytometry

Anti-ICAM-1 (CD54; clone, HA58), -VCAM-1 (CD106; clone, STA) and -CD80 (clone, 2D10.4) (1:50 dilution; all eBioscience) mouse monoclonal antibodies (mAbs) conjugated with PE were used for the flow cytometry. Mouse immunoglobulin G isotype control mAbs were additionally purchased from eBioscience. The flow cytometer (FACSCalibur; BD Biosciences) utilized was equipped with an argon laser tuned at 488 nm, and mean fluorescence intensity (MFI) was measured; all FACS data are expressed as the MFI.

#### Statistical analysis

All data are presented as the mean ± standard deviation. Data were analyzed using a one-way analysis of variance followed by Fisher's least significant difference post hoc test. Calculations were performed using PASW® Statistics version 18 (IBM SPSS, Armonk, NY, USA). P<0.05 was considered to indicate a statistically significant difference.

## Results

### 

#### Effects of quercetin and taraxasterol on cell viability

**Figure 1.**
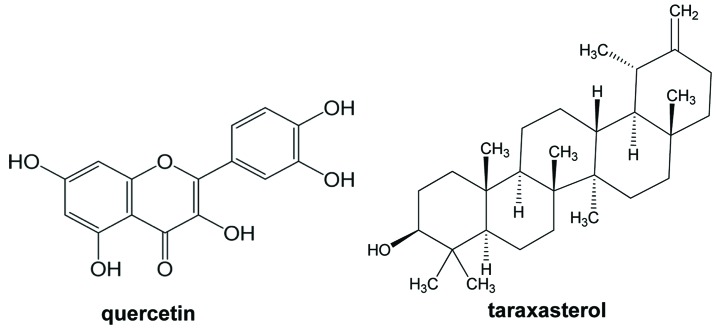
Chemical structures of taraxasterol and quercetin.

**Figure 2.**
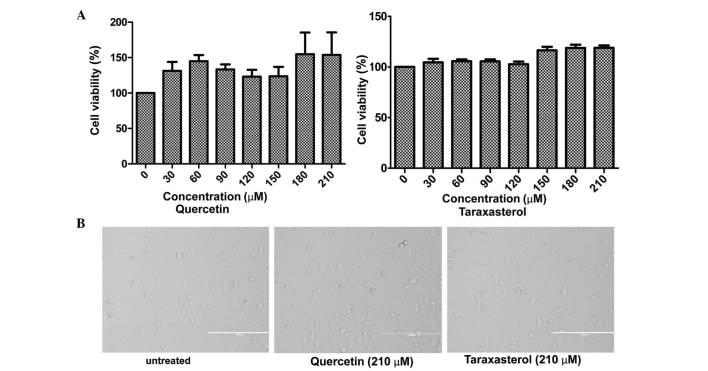
Quercetin and taraxasterol enhance HUVEC viability. (A) HUVECs were treated with different concentrations (30, 60, 90, 120, 150, 180 or 210 µM) of quercetin and taraxasterol for 12 h. Control values were obtained in the absence of quercetin and taraxasterol, and were taken as 100% viability. Data are presented as the mean ± standard deviation of three independent experiments. (B) HUVECs were treated with 210 µM quercetin or taraxasterol for 12 h prior to being visualized by light microscopy and images being captured (original magnification, x200). Untreated HUVECs were used as normal controls. Data were obtained from three independent experiments. HUVEC, human umbilical cord endothelial cell.

Quercetin is a well-known flavonoid, while taraxasterol, a pentacyclic-triterpene, is isolated from *Taraxacum officinale* (Fig. 1). The potential cytotoxicity of quercetin and taraxasterol was evaluated by CCK-8 assay. HUVECs were incubated with quercetin and taraxasterol, respectively, at concentrations ranging between 0 and 210 µM (0, 30, 60, 90, 120, 150, 180 and 210 µM) for 12 h. Compared with untreated cells, the HUVEC morphology was not affected and the viability was not reduced by treatment with either agent (Fig. 2A); however, the agents both stimulated the growth and proliferation of the HUVECs. Of the two compounds, the pro-proliferative effect of quercetin was stronger than that of taraxasterol. The representative microphotographs are shown in Fig. 2B.

#### H_2_O_2_ negatively affects HUVEC viability

**Figure 3.**
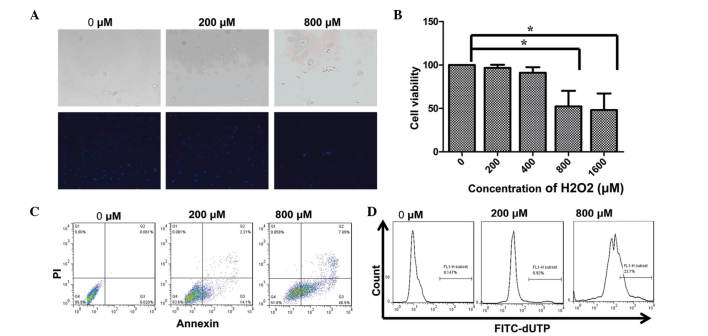
H_2_O_2_-induced apoptosis in HUVECs. (A) Following exposure to 200 µM or 800 µM H_2_O_2_ for 4 h, HUVECs were fixed and labeled with 4′,6′-diamidino-2-phenylindole prior to their observation under an epifluorescence microscope. (B) HUVECs were incubated with different concentrations of H_2_O_2_ for 4 h, and cell viability was determined using a cell counting kit-8 assay. Data are representative of the average of three independent experiments. The cell viability of HUVECs not treated with H_2_O_2_ was taken as the control (100%). Marked cytotoxicity of H_2_O_2_ was shown at concentrations of ≥800 µM. (C and D) Detection of apoptosis in HUVECs using (C) an annexin V/PI staining assay and (D) TUNEL assay. Data are shown for the negative control (not treated with H_2_O_2_) and 200 and 800 µM H_2_O_2_-treated HUVECs. Bars indicate the cells labeled as TUNEL-positive cells. HUVEC, human umbilical cord endothelial cell; PI, propidium iodide; TUNEL, terminal deoxynucleotidyl transferase dUTP nick end labeling; FITC, fluorescein isothiocyanate.

The effect of H_2_O_2_ on HUVECs is shown in Fig. 3. HUVECs were exposed to different doses of H_2_O_2_ (0, 200, 400, 800 or 1,600 µM) for 4 h, and the cell viability was then detected using CCK-8 assay. As shown in Fig. 3B, cell viability was not changed at low H_2_O_2_ concentrations of 200 and 400 µM. At higher concentrations of H_2_O_2_, cell viability was significantly decreased. At H_2_O_2_ concentrations of 800 and 1,600 µM, the cell viability was reduced to 52.34±31.06 and 48.15±32.92%, respectively; therefore, a concentration of 800 µM was used to induce cell injury in subsequent experiments. The results of the DAPI staining reflected the findings of the cell viability assay (Fig. 3A). In cells not treated with H_2_O_2_, no signs of morphological nuclear damage were observed; by contrast, the H_2_O_2_-treated cells exhibited changes in nuclear and actin structure. At a concentration of 800 µM, nuclear breakdown was evident from small fragments of nuclei inside the cells.

#### H_2_O_2_ induces apoptosis in HUVECs

It is well known that H_2_O_2_ can cause lipid peroxidation and DNA damage, which can induce apoptosis. In the present study, two assays were applied to examine the changes in the levels of apoptosis following H_2_O_2_-induced HUVEC injury. As shown by the annexin V/PI staining in Fig. 3C, the percentage of early apoptotic (annexin V^+^/PI^−^) and late apoptotic (annexin V^+^/PI^+^) untreated HUVECs was 0.020 and 0.061%, respectively. By contrast, 4 h of treatment with different concentrations of H_2_O_2_ largely increased the percentage of early and late apoptotic cells. At a concentration of 200 µM, the percentage of early and late apoptotic cells increased to 14.4 and 2.21%, respectively, and at the concentration of 800 µM, these percentages increased to 40.5 and 7.69%. As shown by TUNEL assay in Fig. 3D, comparisons with untreated HUVECs revealed the percentage of TUNEL-positive cells in the HUVECs treated with 200 and 800 µM H_2_O_2_ to be 6.97 and 23.7%, respectively; however, the combined percentages of annexin V^+^/PI^−^ and annexin V^+^/PI^+^ cells were 16.31 and 48.19%, respectively. These values were approximately two-fold higher than the values determined by TUNEL assay. This indicated that annexin V/PI staining was more sensitive than TUNEL assay, as reported by Shen *et al* (26).

#### Quercetin and taraxasterol protect HUVECs against H_2_O_2_-induced cytotoxicity

**Figure 4.**
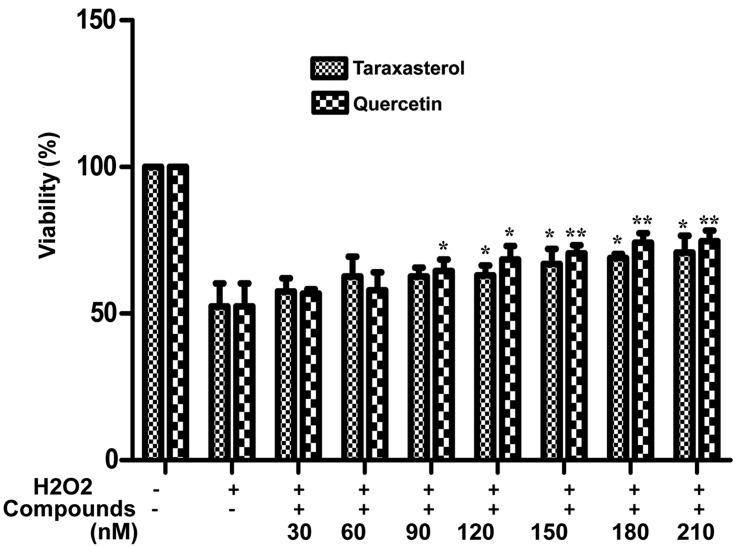
Pretreatment of HUVECs with quercetin and taraxasterol inhibits H_2_O_2_-induced cell injury. HUVECs were pretreated with or without different concentrations of quercetin and taraxasterol individually for 12 h, and then incubated with or without 800 µM H_2_O_2_ for 4 h. The cell viability was evaluated using a cell counting kit-8 assay. *P<0.05 and **P<0.01 versus the cells treated only with H_2_O_2_. HUVEC, human umbilical cord endothelial cell.

Following the apoptosis experiments, the optimal concentrations of quercetin and taraxasterol for inducing the proliferation of HUVECs without altering cell morphology were determined. The protective effects of the two compounds against H_2_O_2_-induced HUVECs injury were then compared. The effects of quercetin and taraxasterol on the viability of H_2_O_2_-treated HUVECs were analyzed by CCK-8 assay. As shown in Fig. 4, treatment with 800 µM H_2_O_2_ for 4 h caused significantly decreased cell viability (~50%); however, pretreatment with different concentrations of taraxasterol (30, 60, 90, 120, 150, 180 or 210 µM) for 12 h significantly improved the viability of the HUVECs (up to 70.78±5.72% viability) and pretreatment with quercetin significantly increased the viability of the HUVECs to a maximum of 74.74±6.12% at 210 µM (P<0.01). The inhibitory effect of quercetin on H_2_O_2_-induced HUVEC injury was stronger than that of taraxasterol at concentrations of 90–210 µM, which is consistent with previous research (27–29). The data also showed that the vascular protective effects of quercetin and taraxasterol against oxidative damage occurred in a dose-dependent manner.

#### VCAM-1 and CD80 are involved in the protection of endothelial cells

Based on the above observation and the anti-inflammatory effects of the two compounds (26–30), we speculated whether quercetin and taraxasterol would affect the expression of pro-inflammatory CAMs and co-stimulatory molecules. Previous research has indicated that selectins mediate the initial rolling of leukocytes along the endothelium and that VCAM-1 and ICAM-1 play important roles in the firm attachment and transendothelial migration of the leukocytes (31). The next focus of the study was therefore on the surface expression of the adhesive molecule VCAM-1 and the co-stimulatory molecule CD80.

**Figure 5.**
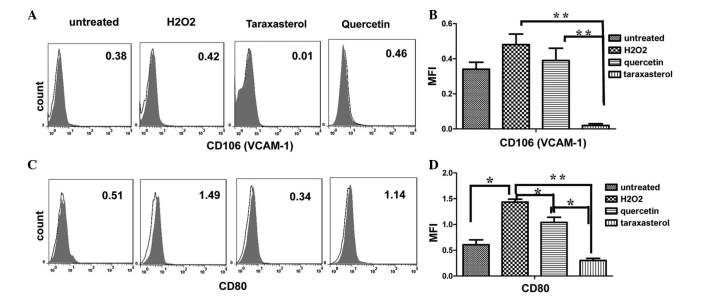
Expression of (A and B) VCAM-1 and (C and D) CD80 on HUVECs. HUVECs were pretreated with 210 µM quercetin or taraxasterol for 12 h individually or left unretreated, and then stimulated with 800 µM H_2_O_2_ for 4 h. HUVECs without quercetin or taraxasterol pretreatment and stimulation of H_2_O_2_ were used as a normal control. Isotypic controls are presented as empty histograms and specific antibody labeling is shown as shaded histograms. Histograms obtained with cells incubated with specific antibodies that overlapped with the isotypic control are shown. (A and C) Cell surface expression of (A) VCAM-1 and (C) CD80 was analyzed by flow cytometry. Both quercetin and taraxasterol reduced the expression of CD80 on activated HUVECs. Data were obtained from three independent experiments. (B and D) The expression of (B) VCAM-1 and (D) CD80 on activated HUVECs was measured in MFI. Error bars represent the mean ± standard error of the mean of two separate experiments. *P<0.05 and **P<0.01. HUVEC, human umbilical cord endothelial cell; VCAM-1, vascular cell adhesion molecule 1; CD, cluster of differentiation; ICAM-1, intracellular adhesion molecule 1.

Although VCAM-1 was not constitutively expressed by HUVECs, it was markedly induced following the stimulation of the HUVECs by 800 µM H_2_O_2_ for 4 h. The MFI increased from 0.34±0.057 to 0.48±0.085. Pretreatment of the HUVECs with quercetin or taraxasterol for 12 h prior to the stimulation with 800 µM H_2_O_2_ for 4 h decreased the expression of VCAM-1. Pretreatment with 210 µM quercetin for 12 h decreased the expression of VCAM-1 to normal levels (0.39±0.099) or lower, and pretreatment with taraxasterol significantly reduced the MFI from 0.48±0.085 to 0.02±0.014 (P<0.01) (Fig. 5A and B).

Having observed that taraxasterol and quercetin reduced the expression of VCAM-1, it was further investigated whether taraxasterol and quercetin could change the expression of co-stimulatory molecules. Due to the fact that deficiencies in the co-stimulatory molecules CD80 and CD86 have been shown to reduce atherosclerosis in mice (32), the surface expression of CD80 molecule on HUVECs was evaluated in the present study by assessing the MFI. As shown in Fig. 5C and D, the expression of CD80 was low in resting HUVECs (0.60±0.13); however, when the HUVECs were activated by H_2_O_2_, the expression of CD80 increased significantly by three-fold. Although taraxasterol and quercetin both reduced the CD80 expression on activated HUVECs, the inhibitory effect exerted by taraxasterol on the enhancement of CD80 expression was more marked (from 1.44±0.77 to 0.30±0.056).

## Discussion

Oxidative stress can result from a variety of external stimuli, including toxins, cytokines, neurohormones, ischemia or mechanical stress (33). It has previously been suggested that an increased formation of reactive oxygen species (ROS) (known as oxidative stress) promotes smooth muscle cell proliferation and matrix formation, and participants in long-term changes in the cardiac cellular phenotype, as observed in hypertrophy, heart failure and apoptosis (34). It is therefore believed that increased ROS production may be partly responsible for a variety of different diseases, such as hypertension (35), restenosis and arteriosclerosis (36). Initially, redox processes were suggested to be mediated predominantly by H_2_O_2_. H_2_O_2_ can modify LDL into oxidized-LDL, which may promote the development of atherosclerosis (37). In the present study, the construction of an H_2_O_2_-induced cell injury model was used to demonstrate that oxidative damage could alter the morphology and function of endothelial cells.

At concentrations of 30–210 µM, both taraxasterol and quercetin increased the viability and proliferation of the HUVECs. At concentrations of 90–210 µM, the inhibitory effect of quercetin on H_2_O_2_-induced HUVEC injury was stronger than that of taraxasterol, and a dose-dependent effect was observed. There are several mechanisms by which taraxasterol and quercetin can protect against oxidative stress-induced epithelial and endothelial cell dysfunction (38,39). A previous study reported that quercetin inhibited the upregulation of caveolin-1 to result in an increase in antioxidative capacity (39). Furthermore, the chronic administration of quercetin has been shown to significantly attenuate elevations in lipid peroxidation and restore depletions in reduced glutathione levels, acetylcholinesterase activity and nitrite activity (27). Taraxasterol has been suggested to exert its anti-inflammatory effect by blocking the nuclear factor-κB (NF-κB) pathway (24).

It is well known that H_2_O_2_ induces apoptosis in HUVECs (40). The findings of the present study also confirmed that H_2_O_2_ could increase the level of HUVEC apoptosis. Comparisons with untreated HUVECs revealed that the percentage of TUNEL-positive cells among the cells treated with 200 and 800 µM H_2_O_2_ was 6.97 and 23.7%, respectively. Furthermore, annexin V/PI staining was found to be a more sensitive method of assessment than TUNEL assay.

In addition to inducing apoptosis, H_2_O_2_ is a powerful pro-inflammatory factor acting in endothelial cells. In a previous study, 2 h incubation with H_2_O_2_ increased the release of tumor necrosis factor-α and IL-6 to 2.67- and 1.67-fold that of control levels, respectively (41). To further confirm the ability of taraxasterol and quercetin to prevent atherogenesis, the current study investigated the effect of taraxasterol and quercetin on the expression of ICAM-1 and VCAM-1 in H_2_O_2_-induced HUVECs. Leukocyte adherence and transmigration across the vascular endothelium are mediated by CAMs (42). Increased levels of ICAM-1 and VCAM-1 have been associated with the early events of atherosclerosis. Quercetin, a bioflavonoid commonly present in a variety of plants, is known to have several biological effects and exerts anti-inflammatory, antioxidant (39), antiviral and antitumor activities. The anti-inflammatory effect of quercetin has received particular focus. In a previous study involving human atheroma, high levels of ICAM-1 expression were found in endothelial cells and macrophages, whereas VCAM-1 expression was observed in fewer than one-third of lesions and its expression was predominantly restricted to endothelial cells (43).

One of the main findings of the present study was that short-term exposure (4 h) of HUVECs to H_2_O_2_ resulted in the induction of the expression of the adhesive molecule VCAM-1 and the co-stimulatory molecule CD80 on HUVECs *in vitro*. Quercetin reduced the expression of VCAM-1 on H_2_O_2_-injured HUVECs in a non-significant manner (P=0.522). This finding was not consistent with that of Rendig *et al* (44), which documented that quercetin inhibited H_2_O_2_-induced endothelium-independent coronary vasorelaxation and significantly downregulated NF-κB, activator protein-1, IL-6 and VCAM-1. Taraxasterol, a pentacyclic-triterpene isolated from *Taraxacum officinale*, *Cichorium glandulosum*, *Cynara cardunculus* L., *Arnica montana* L., *Arctium* and *Cichorium intybus* (45), has various biological activities and exerts antimicrobial (46), antiviral (47,48), anti-inflammatory (24) and chemopreventive (38) effects. In the present study, we found that taraxasterol significantly reduced the expression of VCAM-1 on H_2_O_2_-injured HUVECs. Furthermore, both taraxasterol and quercetin significantly reduced the expression of CD80 on H_2_O_2_-injured HUVECs. A combination of these two compounds may attenuate the inflammatory state in atherosclerosis. These findings demonstrated the beneficial effect of taraxasterol and quercetin in reducing VCAM-1 and CD80 expression on endothelial cells and thus in reducing the risk of atherosclerosis.

**Figure 6.**
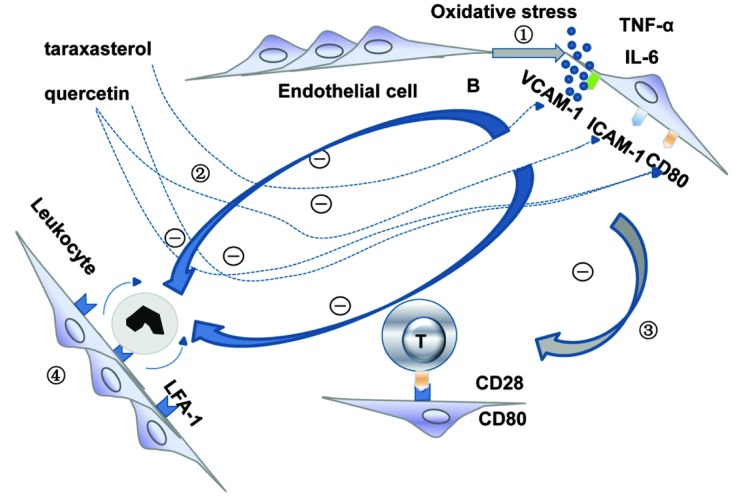
Possible protective mechanism of taraxasterol and quercetin against oxidative stress-induced injury. Step 1: H_2_O_2_ increases the release of TNF-α and IL-6 from HUVECs and upregulates the expression of the adhesive molecule VCAM-1 and the co-stimulatory molecule CD80. Step 2: Pretreatment of the HUVECs with taraxasterol or quercetin inhibits the expression of ICAM-1, VCAM-1 and CD80 on the activated HUVECs. Step 3: A decrease in the expression of CD80 results in the inactivation of T cells. Step 4: A decrease in the expression of ICAM-1 and VCAM-1 results in a reduction of leukocyte rolling. The inactivation of T cells and reduced leukocyte rolling are associated with the progression of atherosclerosis. TNF-α, tumor necrosis factor-α; IL-6, interleukin 6; HUVEC, human umbilical cord endothelial cell; VCAM-1, vascular cell adhesion molecule 1; CD, cluster of differentiation; ICAM-1, intracellular adhesion molecule 1.

In conclusion, as a powerful pro-inflammatory factor, H_2_O_2_ is known to increase the release of TNF-α and IL-6 from HUVECs, on which the adhesive molecule VCAM-1 and the co-stimulatory molecule CD80 are expressed (49). Pretreatment of the HUVECs with taraxasterol or quercetin inhibited the expression of VCAM-1 and CD80 on the activated HUVECs. Decreased expression of CD80 has been found to result in the inactivation of T cells (50), while decreased expression of ICAM-1 results in a reduction in leukocyte rolling (51); both are associated with the progression of atherosclerosis (Fig. 6). The results of the present study showed the protective effects of quercetin and taraxasterol against cell injury and in downregulating the expression of VCAM-1 and CD80 on HUVECs. This study may provide preliminary data for further investigations into the mechanism underlying the anti-atherosclerotic and cardiovascular protective effects of quercetin and taraxasterol as dietary supplements.
